# Approximation of functions in the generalized Zygmund class using Hausdorff means

**DOI:** 10.1186/s13660-017-1361-8

**Published:** 2017-05-05

**Authors:** Mradul Veer Singh, ML Mittal, BE Rhoades

**Affiliations:** 1 Department of Mathematics, University of Petroleum and Energy Studies, Bidholi Campus, Dehradun, Uttarakhand, 248007 India; 2 Department of Mathematics, Indian Institute of Technology, Roorkee, Uttarakhand, 247667 India; 3 Department of Mathematics, Indiana University, Bloomington, IN 47405-7106 USA

**Keywords:** 42A10, 42A25, 40C05, Zygmund class, degree of approximation, trigonometric Fourier series, Hausdorff means

## Abstract

In this paper we investigate the degree of approximation of a function belonging to the generalized Zygmund class $Z_{p}^{(\omega)}$ ($p \ge1$) by Hausdorff means of its Fourier series. We also deduce a corollary and mention a few applications of our main results.

## Introduction

During the last few decades the degree of approximation of functions belonging to various Lipschitz classes (Lip*α*, $\operatorname{Lip}(\alpha, p)$, $\operatorname{Lip}(\xi(t), p)$, $W(L_{p}, \xi(t))$ etc.) has been studied by various investigators (see [1–11] and the references therein) using different summability methods such as Cesàro, Hölder, Euler and their products. Each of the matrices involved in these methods is a Hausdorff matrix and the product of two Hausdorff matrices is again a Hausdorff matrix [12–15]. Also, multiplication of two Hausdorff matrices is commutative. Thus in view of these remarks, Rhoades [16–19], Singh, Srivastava [20] have obtained the degree of approximation of functions belonging to various Lipschitz classes using Hausdorff means. To the best of our knowledge, the approximation of functions belonging to the generalized Zygmund class by Hausdorff means has not been investigated so far. This motivated us to work in this direction. Here we recall some definitions for our purpose.

Let $L_{p}:= L_{p} [0, 2 \pi] = \{f: [0, 2\pi] \to\mathbb{R}; \int _{0}^{2 \pi} \vert f(x) \vert ^{p} \,dx < \infty\}$, $p \ge1$ be the space of all 2*π*-periodic integrable functions. The $L_{p}$-norm of such a function *f* ([21], p.36) is defined by

$$ \Vert f \Vert _{p} := \textstyle\begin{cases} (\frac{1}{2\pi} \int_{0}^{2\pi} \vert f(x) \vert ^{p} \,dx )^{1/p}, & 1 \le p < \infty; \\ \operatorname{ess} \sup_{0 < x \le2 \pi} \vert f(x) \vert , & p=\infty. \end{cases} $$

For a given function $f \in L_{p}$, let

1.1 $$ s_{n}(f):= s_{n}(f;x)= \frac{a_{0}}{2}+\sum _{k=1}^{n} (a_{k} \cos kx+b_{k} \sin kx )= \sum_{k=0}^{n} u_{k}(f;x) $$

denote the partial sums, called trigonometric polynomials of degree (or order) *n*, of the first $(n+1)$ terms of the Fourier series of *f* at a point *x*.

### Note 1

Here we shall use the notations of Lal et al. [22].

Let $\omega:[0, 2 \pi] \to\mathbb{R}$ be an arbitrary function with $\omega(t)>0$ for $0 < t \le2\pi$ and $\lim_{t \to0^{+}} \omega(t) = \omega(0) = 0$. As in [22], define

$$Z_{p}^{(\omega)}:=\biggl\{ f \in L_{p} : 1 \le p < \infty, \sup_{t \neq0} \frac{ \Vert f(\cdot+t)+f(\cdot-t)-2f(\cdot) \Vert _{p}}{\omega (t)} < \infty\biggr\} $$

and

$$\Vert f \Vert _{p}^{(\omega)}:= \Vert f \Vert _{p} + \sup_{t \neq0} \frac{ \Vert f(\cdot+t)+f(\cdot-t)-2f(\cdot) \Vert _{p}}{\omega(t)}, \quad p \ge1. $$

The completeness of $Z_{p}^{(\omega)}$ can be discussed by considering the completeness of $L_{p}$ ($p \ge1$), and hence the space $Z_{p}^{(\omega )}$ is a Banach space under the norm $\Vert \cdot \Vert _{p}^{(\omega)}$.

Some other forms of the generalized Zygmund classes have been investigated by Leindler [23], Móricz and Németh [24] and Móricz [25]. Throughout this paper *ω* and *v* will denote the Zygmund moduli of continuity such that $\omega(t)/v(t)$ is positive and non-decreasing in *t*. For well-known properties of modulus of continuity and modulus of smoothness, in our case Zygmund modulus of continuity, one can refer the excellent monograph of Zygmund ([26], pp.42-45).

Note that

$$\Vert f \Vert _{p}^{(v)} \le\max \biggl( 1, \frac{\omega(2 \pi )}{v(2 \pi)} \biggr) \Vert f \Vert _{p}^{(\omega)} < \infty. $$

Thus we have $Z_{p}^{(\omega)} \subseteq Z_{p}^{(v)} \subseteq L_{p}$, $p \ge1$.

### Note 2

(i) If $\omega(t)= t^{\alpha}$, then the $Z^{(\omega)}$ and $Z^{(\omega )}_{p}$ classes reduce to the $Z_{\alpha}$ and $Z_{\alpha, p}$ classes, respectively.

(ii) If we take $p \to\infty$, then the $Z^{(\omega)}_{p}$ class reduces to the $Z^{(\omega)}$ class and the $Z_{\alpha, p}$ class reduces to the $Z_{\alpha}$ class.

(iii) Let $0 \le\beta<\alpha<1$. If $\omega(t)= t^{\alpha}$ and $v(t)= t^{\beta}$, then, clearly, $\omega(t)/v(t)$ is non-decreasing, while $\omega(t)/t v(t)$ is a non-increasing function of *t*.

## Preliminaries

Hausdorff matrices were first introduced by Hurwitz and Silverman (1917) as the collection of lower triangular matrices that commute with the Cesàro matrix of order one. Hausdorff (1921) reintroduced this class in the process of solving the moment problem over a finite interval [27]. A Hausdorff matrix $H \equiv(h_{n,k})$ is an infinite lower triangular matrix [13] defined by

$$h_{n, k}= \textstyle\begin{cases} {n \choose k} \triangle^{n-k} \mu_{k} , & 0\le k \le n; \\ 0, & k>n, \end{cases} $$

where the operator △ is defined by $\triangle\mu_{n} \equiv \mu_{n}-\mu_{n+1}$ and $\triangle^{k+1} \mu_{n} \equiv\triangle^{k} (\triangle\mu_{n})$.

A Hausdorff matrix is regular iff $\int_{0}^{1} \vert \, d \gamma (u) \vert < \infty$, where the mass function $\gamma(u)$ is continuous at $u=0$ and belongs to $BV[0, 1]$ such that $\gamma(0)=0$, $\gamma(1)=1$; and for $0< u<1$, $\gamma(u)= [\gamma(u+0)+\gamma (u-0)]/2$. In this case $\{\mu_{n}\}$ has the representation $\mu_{n}=\int _{0}^{1} u^{n}\, d \gamma(u)$. A detailed discussion and further interesting properties of Hausdorff matrices can be seen in [13–15, 27, 28] and the references given there.

The Hausdorff means of a trigonometric Fourier series of *f* is defined by

$$H_{n}(x):= H_{n}(f, x)= \sum_{k=0}^{n} h_{n, k} s_{k}(f, x), \quad \forall n \ge0. $$

The Fourier series of a function *f* is said to be summable to *s* by Hausdorff means if $H_{n}(x) \to s$ as $n \to\infty$.

We need the following lemmas in the proofs of our theorems.

### Lemma 1

*Let* $g(u, t):= \operatorname{Im} [\sum_{k=0}^{n} {n \choose k} u^{k} (1-u)^{n-k} e^{i(k+(1/2))t} ]$ *for* $0\le u\le1$ *and* $0 \le t \le\pi$. *Then*

$$\biggl\vert \int_{0}^{1} g(u, t)\, d \gamma(u) \biggr\vert = \textstyle\begin{cases} O((n+1)t) , & 0\le t \le1/(n+1); \\ O((n+1)^{-1} t^{-1}), & 1/(n+1) \le t \le\pi. \end{cases} $$

### Proof

We can write

$$ g(u, t)= (1-u)^{n} \operatorname{Im} \Biggl[e^{it/2} \sum _{k=0}^{n} {n \choose k} \biggl( \frac{u e^{it}}{1-u} \biggr)^{k} \Biggr] = \operatorname {Im} \bigl[e^{it/2}\bigl(1-u+ue^{it}\bigr)^{n} \bigr], $$

which is continuous for $u \in[0, 1]$. If $M = \sup_{0\le u \le1} \frac{d \gamma(u)}{du}$ ([20], p.636), then

$$\begin{aligned} \int_{0}^{1} g(u, t)\, d \gamma(u) &\le M \int_{0}^{1} g(u, t) \,du \\ &= O(1) \int_{0}^{1} \operatorname{Im} \bigl[e^{it/2}\bigl(1-u+ue^{it}\bigr)^{n} \bigr] \,du \\ &=O(1) \operatorname{Im} \biggl[ \frac {(1-u+ue^{it})^{n+1}}{(e^{it/2}-e^{-it/2}) (n+1)} \biggr]_{u=0}^{1} \\ &= O(1) \operatorname{Im} \biggl[ \frac{e^{i(n+1)t} -1}{(e^{it/2}-e^{-it/2}) (n+1)} \biggr] \\ &=O(1) \operatorname{Im} \biggl[ \frac{e^{i(n+1)t} -1}{2 i \sin(t/2) (n+1)} \biggr] = O \biggl( \frac{1-\cos(n+1)t}{2(n+1)\sin(t/2)} \biggr) \\ &= O \biggl(\frac{\sin^{2} (n+1)t/2}{(n+1)\sin(t/2)} \biggr). \end{aligned}$$

*Case* (i): For $0\le t \le1/(n+1)$, we have

$$\biggl\vert \int_{0}^{1} g(u, t)\, d \gamma(u) \biggr\vert = O \bigl((n+1)t\bigr), $$

since $\vert \sin kt \vert \le k \vert \sin t \vert $ $\forall k \in\mathbb{N}$.

*Case* (ii): For $1/(n+1) \le t \le\pi$, we have

$$\biggl\vert \int_{0}^{1} g(u, t)\, d \gamma(u) \biggr\vert = O \bigl((n+1)^{-1}t^{-1}\bigr), $$

using $\vert \sin t \vert \le1$ for all *t* and $(\sin t)^{-1} \le\pi/2t$ for $0 < t \le\pi/2$. This completes the proof of Lemma 1. □

### Lemma 2

*Let* $K_{n}^{H}(t):=[2 \pi\sin(t/2)]^{-1} \int _{0}^{1} g(u,t) \,d\gamma(u)$. *Then*

$$\bigl\vert K_{n}^{H}(t) \bigr\vert = \textstyle\begin{cases} O(n+1) , & 0\le t \le1/(n+1); \\ O((n+1)^{-1} t^{-2}), & 1/(n+1) \le t \le\pi. \end{cases} $$

### Proof

The proof follows directly using $(\sin t)^{-1} \le\pi/2t$ for $0 < t \le\pi/2$ and Lemma 1. □

Analogous to Lemma 1 of Das et al. [29], Lal and Shireen [22] have proved the following.

### Lemma 3

[22], p. 8

*Let* $f \in Z_{p}^{(\omega)}$, *then for* $0 < t \le\pi$,

(i) $\Vert \phi(\cdot, t) \Vert _{p} = O(\omega(t))$.
(ii) $\Vert \phi(\cdot+y, t) + \phi(\cdot-y, t)- 2\phi(\cdot, t) \Vert _{p} = \begin{cases} O(\omega(t)), \\ O(\omega(y)). \end{cases} $
(iii) *If* $\omega(t)$ *and* $v(t)$ *are defined as in Theorem* 1, *then*
  $$\bigl\Vert \phi(\cdot+y, t) + \phi(\cdot-y, t)- 2\phi(\cdot, t) \bigr\Vert _{p} = O \biggl( v(y) \frac{\omega(t)}{v(t)} \biggr), $$
  *where* $\phi(x, t) = f(x+t)+f(x-t)-2f(x)$.

## Main result and discussion

Fundamental physical quantities such as electro-magnetic field, pressure, voltage-change in time are called time waveforms or signals [30]. The Fourier approximation of signals (functions) that originated from the second theorem of Weierstrass has become an exciting interdisciplinary field of research for the last 130 years. These approximations have assumed important new dimensions due to their wide applications in signal analysis [31], in general and in digital signal processing [32] in particular, in view of the classical Shannon sampling theorem [7].

In this paper we prove two theorems on approximation of a function by Hausdorff means of its Fourier series in terms of Zygmund modulus of continuity.

### Theorem 1

*Let* *f* *be a* 2*π* *periodic function*, *Lebesgue integrable on* $[-\pi, \pi]$ *and belonging to the generalized Zygmund class* $Z_{p}^{(\omega )}$, $p \ge1$. *Then the degree of approximation of a signal* (*function*) *f*, *using Hausdorff means of its Fourier series*, *is given by*

3.1 $$ E_{n}(f)= \inf_{n} \Vert H_{n}-f \Vert _{p}^{(v)} = O \biggl( (n+1)^{-1} \int_{(n+1)^{-1}}^{\pi} \frac{t^{-2} \omega(t)}{ v(t)} \,dt \biggr), $$

*where* *ω* *and* *v* *denote the Zygmund moduli of continuity such that* $\omega(t)/v(t)$ *is positive and non*-*decreasing*.

### Theorem 2

*In addition to the conditions of Theorem* 1, *if* $t^{-1} \omega (t)/v(t)$ *is non*-*increasing*, *then the degree of approximation of a signal* (*function*) *f* *in* $Z_{p}^{(\omega)}$ ($p \ge1$), *using Hausdorff means of its Fourier series*, *is given by*

3.2 $$ E_{n}(f)= O \biggl( \frac{\omega((n+1)^{-1})}{v((n+1)^{-1})} \log(n+1) \biggr). $$

Now, in view of Note 2, we present a corollary of our results.

### Corollary 1

*Let* *f* *be a* 2*π* *periodic function*, *Lebesgue integrable on* $[-\pi, \pi]$ *and belonging to the Zygmund class* $Z_{\alpha, p}$, $p \ge1$. *Then the degree of approximation of a signal* (*function*) *f*, *using Hausdorff means of its Fourier series*, *is given by*

$$ E_{n}(f)= \inf_{n} \Vert H_{n}-f \Vert _{p}^{(\beta)} = \textstyle\begin{cases} O ((n+1)^{\beta-\alpha} ) , & 0 \le\beta< \alpha< 1; \\ O ((n+1)^{-1} \log(n+1) ), & \beta=0, \alpha=1. \end{cases} $$

### Proof

The result can be obtained by putting $\omega(t)= t^{\alpha}$ and $v(t)= t^{\beta}$ in Theorems 1 and 2. □

### Remark 1

We observe that these results are analogous to those of Lal and Shireen [22]. Similar estimates for Hölder classes have been discussed by Das et al.[29] and Leindler [33].

### Remark 2

The method $(C, k)$ is a Hausdorff method corresponding to the mass function $\gamma(u)= k \int_{0}^{u} (1-t)^{k-1} \,dt$. The Hölder method $(H, k)$ is a Hausdorff method corresponding to the mass function, $\gamma(u)= \int_{0}^{u} \frac{1}{(k-1)!} (\log(1/t))^{k-1} \,dt$ ([12], pp.39-40). For the mass function

$$\gamma(u)= \textstyle\begin{cases} 0, & \mbox{if }0 \le u < a; \\ 1, & \mbox{if }a \le u \le1, \end{cases} $$

where $a= 1/(1+q)$, $q>0$, it is easy to verify that $\mu _{k}=1/(1+q)^{k}$ and

$$h_{n, k}= \textstyle\begin{cases} {n \choose k} q^{n-k} (1+q)^{-n}, & \mbox{if }0 \le k \le n; \\ 0, & \mbox{if }k > n. \end{cases} $$

Thus the Hausdorff matrix $H \equiv(h_{n, k})$ reduces to the Euler matrix $(E, q)$ of order $q >0$.

In view of the above remark, our results also hold for different summability methods such as Cesàro, Hölder, Euler and their products.

## Proof of main result

### Proof of Theorem 1

It is well known [26] that

4.1 $$ s_{k}(f,x)- f(x)= \frac{1}{2 \pi} \int_{0}^{\pi} \phi(x, t) \frac{\sin (k+2^{-1})t}{\sin(t/2)} \,dt. $$

Since $\sum_{k=0}^{n} h_{n, k} = 1$ for each *n* in any regular Hausdorff matrix *H* ([13], p.397), so we have

$$\begin{aligned} H_{n}(x)-f(x) &= \frac{1}{2 \pi} \int_{0}^{\pi} \phi(x, t) \sum _{k=0}^{n} h_{n, k} \frac{\sin(k+2^{-1})t}{\sin(t/2)} \,dt \\ &= \frac{1}{2 \pi} \int_{0}^{\pi} \frac{\phi(x, t)}{\sin(t/2)} \sum _{k=0}^{n} \int_{0}^{1} {n \choose k} u^{k} (1-u)^{n-k}\, d \gamma(u) \operatorname{Im} \bigl(e^{i(k+2^{-1})t}\bigr) \,dt \\ &= \frac{1}{2 \pi} \int_{0}^{\pi} \frac{\phi(x, t)}{\sin(t/2)} \int _{0}^{1} \operatorname{Im} \Biggl[\sum _{k=0}^{n} {n \choose k} u^{k} (1-u)^{n-k} e^{i(k+2^{-1})t} \Biggr]\, d \gamma(u) \,dt \\ &= \frac{1}{2 \pi} \int_{0}^{\pi} \frac{\phi(x, t)}{\sin(t/2)} \int _{0}^{1} g(u, t)\, d \gamma(u) \,dt. \end{aligned}$$

Let

4.2 $$ l_{n}(x) := H_{n}(x)-f(x)= \int_{0}^{\pi} \phi(x, t) K_{n}^{H}(t) \,dt. $$

Then

$$l_{n}(x+y)+ l_{n}(x-y)-2 l_{n}(x) = \int_{0}^{\pi} \bigl[\phi(x+y, t)+\phi (x-y, t)- 2 \phi(x,t)\bigr] K_{n}^{H}(t) \,dt. $$

Using the generalized Minkowski inequality ([21], p.37), we get

4.3 $$\begin{aligned} & \bigl\Vert l_{n}(\cdot+ y)+ l_{n}(\cdot- y)-2 l_{n}(\cdot) \bigr\Vert _{p} \\ &\quad = \biggl\{ \frac{1}{2 \pi} \int_{0}^{2 \pi} \bigl\vert l_{n}(x+y)+ l_{n}(x-y)-2 l_{n}(x) \bigr\vert ^{p} \,dx \biggr\} ^{1/p} \\ &\quad = \biggl\{ \frac{1}{2 \pi} \int_{0}^{2 \pi} \biggl\vert \int_{0}^{\pi} \bigl[\phi(x+y, t)+\phi(x-y, t)- 2 \phi(x, t)\bigr] K_{n}^{H}(t) \,dt \biggr\vert ^{p} \,dx \biggr\} ^{1/p} \\ &\quad \le \int_{0}^{\pi} \biggl\{ \frac{1}{2 \pi} \int_{0}^{2 \pi} \bigl\vert \bigl[\phi(x+y, t)+ \phi(x-y, t)- 2 \phi(x, t)\bigr] K_{n}^{H}(t) \bigr\vert ^{p} \,dx \biggr\} ^{1/p} \,dt \\ &\quad = \int_{0}^{\pi} \bigl( \bigl\vert K_{n}^{H}(t) \bigr\vert ^{p} \bigr)^{1/p} \biggl\{ \frac{1}{2 \pi} \int_{0}^{2 \pi} \bigl\vert \phi(x+y, t)+\phi(x-y, t)- 2 \phi(x, t) \bigr\vert ^{p} \,dx \biggr\} ^{1/p} \,dt \\ &\quad = \int_{0}^{\pi} \bigl\Vert \phi(\cdot+y, t)+\phi( \cdot-y, t)-2\phi (\cdot, t) \bigr\Vert _{p} \bigl\vert K_{n}^{H}(t) \bigr\vert \,dt \\ &\quad = \int_{0}^{1/(n+1)} \bigl\Vert \phi(\cdot+y, t)+\phi( \cdot-y, t)-2\phi (\cdot, t) \bigr\Vert _{p} \bigl\vert K_{n}^{H}(t) \bigr\vert \,dt \\ &\qquad{} + \int_{1/(n+1)}^{\pi} \bigl\Vert \phi(\cdot+y, t)+\phi( \cdot -y, t)-2\phi(\cdot, t) \bigr\Vert _{p} \bigl\vert K_{n}^{H}(t) \bigr\vert \,dt \\ &\quad := I_{1} + I_{2}, \quad\text{say}. \end{aligned}$$

Using Lemma 2, Lemma 3 {part(iii)} and the monotonicity of $\omega(t)/v(t)$ with respect to *t*, we have

4.4 $$ \begin{aligned}[b] I_{1} &= \int_{0}^{(n+1)^{-1}} \bigl\Vert \phi(\cdot+y, t)+\phi( \cdot-y, t)-2\phi(\cdot, t) \bigr\Vert _{p} \bigl\vert K_{n}^{H}(t) \bigr\vert \,dt \\ &= O \biggl( \int_{0}^{(n+1)^{-1}} v(y) \frac{\omega(t)}{v(t)} (n+1) \,dt \biggr) \\ &= O \biggl((n+1) v(y) \int_{0}^{(n+1)^{-1}} \frac{\omega(t)}{v(t)} \,dt \biggr) \\ &= O \biggl((n+1) v(y) \frac{\omega((n+1)^{-1})}{v((n+1)^{-1})} \int _{0}^{(n+1)^{-1}} \,dt \biggr) \\ &= O \biggl( v(y) \frac{\omega((n+1)^{-1})}{v((n+1)^{-1})} \biggr). \end{aligned} $$

Using Lemma 2 and Lemma 3 {part(iii)}, we get

4.5 $$\begin{aligned} I_{2} &= \int_{(n+1)^{-1}}^{\pi} \bigl\Vert \phi(\cdot+ y, t)+\phi( \cdot - y, t)-2\phi(\cdot, t) \bigr\Vert _{p} \bigl\vert K_{n}^{H}(t) \bigr\vert \,dt \\ &= O \biggl( \int_{(n+1)^{-1}}^{\pi} v(y) \frac{\omega(t)}{v(t)} (n+1)^{-1} t^{-2} \,dt \biggr) \\ &= O \biggl((n+1)^{-1} v(y) \int_{(n+1)^{-1}}^{\pi} \frac{t^{-2} \omega (t)}{v(t)} \,dt \biggr). \end{aligned}$$

Thus, from (4.3), (4.4) and (4.5),

4.6 $$ \begin{gathered} \bigl\Vert l_{n}(\cdot+ y)+l_{n}(\cdot- y)-2l_{n}(\cdot) \bigr\Vert _{p}\\ \quad = O \biggl( v(y) \frac{\omega((n+1)^{-1})}{v((n+1)^{-1})} \biggr) + O \biggl((n+1)^{-1} v(y) \int_{(n+1)^{-1}}^{\pi} \frac{t^{-2} \omega (t)}{v(t)} \,dt \biggr) , \\ \sup_{y \neq0} \frac{ \Vert l_{n}(\cdot+ y)+l_{n}(\cdot- y)-2l_{n}(\cdot) \Vert _{p}}{v(y)}\\ \quad = O \biggl( \frac{\omega ((n+1)^{-1})}{v((n+1)^{-1})} \biggr) + O \biggl((n+1)^{-1} \int _{(n+1)^{-1}}^{\pi} \frac{t^{-2} \omega(t)}{v(t)} \,dt \biggr). \end{gathered} $$

Again using Lemmas 2 and 3, we have

4.7 $$\begin{aligned} \bigl\Vert l_{n}(\cdot) \bigr\Vert _{p} &\le \biggl( \int _{0}^{(n+1)^{-1}} + \int_{(n+1)^{-1}}^{\pi} \biggr) \bigl\Vert \phi(\cdot , t) \bigr\Vert _{p} \bigl\vert K_{n}^{H}(t) \bigr\vert \,dt \\ &= O \biggl((n+1) \int_{0}^{(n+1)^{-1}} \omega(t) \,dt \biggr)+ O \biggl( (n+1)^{-1} \int_{(n+1)^{-1}}^{\pi} t^{-2} \omega(t) \,dt \biggr) \\ &= O \bigl(\omega\bigl((n+1)^{-1}\bigr) \bigr) + O \biggl((n+1)^{-1} \int _{(n+1)^{-1}}^{\pi} t^{-2} \omega(t) \,dt \biggr). \end{aligned}$$

Now, from (4.6) and (4.7), we obtain

4.8 $$ \begin{aligned}[b] \bigl\Vert l_{n}(\cdot) \bigr\Vert _{p}^{(v)} &= \bigl\Vert l_{n}(\cdot ) \bigr\Vert _{p} + \sup_{y \neq0} \frac{ \Vert l_{n}(\cdot+ y)+l_{n}(\cdot- y)-2l_{n}(\cdot) \Vert _{p}}{v(y)} \\ &= O \bigl(\omega\bigl((n+1)^{-1}\bigr) \bigr) + O \biggl((n+1)^{-1} \int _{(n+1)^{-1}}^{\pi} t^{-2} \omega(t) \,dt \biggr) \\ &\quad{}+ O \biggl( \frac{\omega((n+1)^{-1})}{v((n+1)^{-1})} \biggr) + O \biggl((n+1)^{-1} \int_{(n+1)^{-1}}^{\pi} \frac{t^{-2} \omega(t)}{v(t)} \,dt \biggr) \\ &:= \sum_{i=1}^{4} O(J_{i}),\quad \text{say}. \end{aligned} $$

Now we write $J_{1}$ in terms of $J_{3}$ and further $J_{2}$, $J_{3}$ in terms of $J_{4}$.

In view of the monotonicity of $v(t)$, we have

$$\omega(t)= \frac{\omega(t)}{v(t)} v(t) \le v(\pi) \frac{\omega (t)}{v(t)}= O \biggl( \frac{\omega(t)}{v(t)} \biggr)\quad \text{for } 0 < t \le\pi. $$

Hence, for $t=(n+1)^{-1}$,

4.9 $$ J_{1}=O ( J_{3} ). $$

Again by the monotonicity of $v(t)$,

4.10 $$\begin{aligned} J_{2}&= (n+1)^{-1} \int_{(n+1)^{-1}}^{\pi} \frac{t^{-2} \omega (t)}{v(t)} v(t) \,dt \\ &\le(n+1)^{-1} v(\pi) \int_{(n+1)^{-1}}^{\pi} \frac{t^{-2} \omega (t)}{v(t)} \,dt = O (J_{4} ). \end{aligned}$$

Using the fact that ${\omega(t)}/{v(t)}$ is positive and non-decreasing, we have

$$\begin{aligned} J_{4}&=(n+1)^{-1} \int_{(n+1)^{-1}}^{\pi} \frac{t^{-2} \omega(t)}{v(t)} \,dt \\ &\ge\frac{\omega((n+1)^{-1})}{v((n+1)^{-1})} (n+1)^{-1} \int _{(n+1)^{-1}}^{\pi} t^{-2} \,dt = \frac{\omega ((n+1)^{-1})}{v((n+1)^{-1})} (n+1)^{-1} \bigl( n+1 -{\pi}^{-1} \bigr) \\ &\ge\frac{\omega((n+1)^{-1})}{2 v((n+1)^{-1})}, \end{aligned}$$

as $(n+1)^{-1} (n+1-{\pi}^{-1}) > (n+1)^{-1} {n} \ge1/2$. Therefore

4.11 $$ J_{3}= O (J_{4} ). $$

Combining (4.8) with (4.11), we get

$$\bigl\Vert l_{n}(\cdot) \bigr\Vert _{p}^{(v)} = O(J_{4}) = O \biggl((n+1)^{-1} \int_{(n+1)^{-1}}^{\pi} \frac{t^{-2} \omega(t)}{v(t)} \,dt \biggr). $$

Hence

$$E_{n}(f)= \inf_{n} \bigl\Vert l_{n}( \cdot) \bigr\Vert _{p}^{(v)} = O \biggl((n+1)^{-1} \int_{(n+1)^{-1}}^{\pi} \frac{t^{-2} \omega(t)}{v(t)} \,dt \biggr). $$

This completes the proof of Theorem 1.

### Proof of Theorem 2

Following the proof of Theorem 1, we have

$$E_{n}(f)= O \biggl((n+1)^{-1} \int_{(n+1)^{-1}}^{\pi} \frac{t^{-2} \omega (t)}{v(t)} \,dt \biggr). $$

From the assumption that ${t^{-1} \omega(t)}/{v(t)}$ is positive and non-increasing with *t*, we have

$$\begin{aligned} E_{n}(f) &= O \biggl( (n+1)^{-1} (n+1) \frac{\omega ((n+1)^{-1})}{v((n+1)^{-1})} \int_{(n+1)^{-1}}^{\pi} t^{-1} \,dt \biggr) \\ &= O \biggl( \frac{\omega((n+1)^{-1})}{v((n+1)^{-1})} \log(n+1) \biggr). \end{aligned}$$

This completes the proof of Theorem 2.

## Conclusions

Analysis and approximation of signals (or functions) are of great importance in science and engineering because a signal conveys the attribute of some physical phenomenon. Functions in $L_{p}$ ($p \ge 1$)-spaces are assumed to be most appropriate for practical purposes; for example, $L_{1}$, $L_{2}$ and $L_{\infty}$ are of particular interest for engineers in digitization. Fourier methods are commonly used for signal analysis and system design in modern telecommunications, radar and image processing systems. The theory of classical Fourier analysis can be extended to discrete time signals and leads to many effective algorithms that can be directly implemented on general computers or special purpose digital signal processing devices. Thus the study of error estimate of functions in various function spaces such as Lipschitz, Holder, Zygmund, Besov spaces etc. using some summability means of trigonometric Fourier series, also known as trigonometric Fourier approximation (TFA) in the literature, has received a growing interest of investigators over the past few decades. The scientists and engineers use the properties of TFA in designing digital filters. A few more applications have been mentioned in Section 3.

The problem of determining the order of best approximation plays a very important role in approximation theory. In this paper we compute the best approximation of a function *f* in the generalized Zygmund class $Z_{p}^{\omega}$ ($p\ge1$) by Hausdorff means of partial sums of trigonometric Fourier series of *f*. We also deduce a corollary for the $Z_{\alpha, p}$ class. Further we observe that our estimates are analogous to those of Hölder classes. Since, in view of Remark 2, the popular summability methods such as Cesàro, Hölder, Euler and their product methods are particular cases of Hausdorff method, so our results also hold for these methods.

Besov spaces serve to generalize more elementary function spaces and are effective in measuring the smoothness properties of functions. The best approximation of functions in Besov spaces by Hausdorff means may be the future interest of a few investigators in the direction of this work.
